# Knowledge, Attitude, and Practices of Unused Medications Disposal among Patients Visiting Public Health Centers in Gondar Town, Ethiopia: A Cross-Sectional Study

**DOI:** 10.1155/2021/5074380

**Published:** 2021-12-30

**Authors:** Alem Endeshaw Woldeyohanins, Meaza Adugna, Tigabu Mihret, Zemene Demelash Kifle

**Affiliations:** ^1^Department of Social and Administrative Pharmacy, School of Pharmacy, College of Medicine and Health Sciences, University of Gondar, Gondar, Ethiopia; ^2^Department of Pharmacology, School Pharmacy, College of Medicine and Health Sciences, University of Gondar, Gondar, Ethiopia

## Abstract

**Background:**

The improvement of healthcare systems has brought the subsequent increase in people access to medications. The consumers are not able to use all the dispensed medications because of various reasons. The improper disposal of these unused medicines has led to environmental contamination to an alarming extent. Therefore, the study was conducted to assess the knowledge, attitude, and practice of unused medications disposal among patients visiting public health centers in Gondar town, Ethiopia.

**Methods:**

A descriptive cross-sectional study was conducted among 404 patients visiting public health centers in Gondar town, Ethiopia, from August to September 2021, by using standardized and a locally translated semistructured questionnaire. The data were entered and analyzed by using the SPSS version of 21.0. The variable of interest was described in the form of statements and tables.

**Result:**

Out of 404 respondents included in the study, 221 (54.7%) of them were female. More than half (226 (55.9%)) of the respondents had unused medicine at home. The common methods of disposal practice were throwing into a household garbage (150 (31.4%)), followed by flushed into toilet/sinks (88 (21.8%)). More than half (286 (70.8%)) of the respondents knew about medication waste and 239 (59.2%) of them checked their medication expiry date. More than half (215 (53.5%)) of the participants strongly agreed that unused medicine can cause potential risk at home and 279 (69.1%) of study participants strongly agreed that children are more vulnerable to the potential risk of unused medicine at home.

**Conclusion:**

There was a high practice of keeping medication at home, and most disposal approach indicated by the participants was not recommended methods. Awareness about the proper disposal of unused medicines among the public should be created. Guidelines on safe disposal are required, and an organized method of collecting unused and expired pharmaceuticals needs to be introduced.

## 1. Introduction

Medicines are important in saving lives but can cause detrimental effects if inappropriately consumed and managed [1, 2]. The improvement of healthcare systems has brought a subsequent increase in people's access to medications. This increased access has raised the question of how well individual households are well equipped in handling the subsequent rise in medical waste [3].

Patients store their medication at home due to side effects, change of dosage, getting better, and the expiration date [4]. People dispose unused medication improperly into the household trash through landfill and the liquid medications through the sewerage system and these medications are introduced into the environment [5]. Improper drug disposal practice of unused or expired medications is a danger for accidental poisoning and abuse, wasted resources, and can kill aquatic or wildlife and antibiotic resistance [6]. Subsequently, the disposal practice of unused medicines has become a worldwide challenge catching the attention of policymakers, health professionals, pharmaceuticals companies, and the community in general [7].

Globally, safe disposal of expired, unwanted, or unused medications particularly by the consumers is of high concern [8]. Many developed countries have programs aimed at disposal of unused medicines. There has been the National Return and Disposal of Unwanted Medicines Project which is fully supported by the government and pharmaceutical industry [8].

In African countries, programs or systems advocating safe disposal practices of unused medicines are still limited [9]. In Ethiopia, also there is a large accumulation of medication waste due to the unavailability of good pharmaceutical waste management practices, shortage of disposal facilities, poor quality administrative and regulatory system of medicinal waste management, and shortage of clear guidelines [10]. Although a few studies have been done to assess the knowledge, attitude, and disposal practice of unused and expired pharmaceuticals in Ethiopia, there is still a dearth of information regarding the disposal practices for unused medications in both the community and wastage at hospital care settings [11]. There are no studies that report the disposal practices used for unwanted and expired medication in health centers of Gondar town. This triggers the need to conduct the study to assess the knowledge, attitude, and practice of unused medications disposal among patients visiting public health centers in Gondar town, Ethiopia.

## 2. Methods and Materials

### 2.1. Study Design, Study Area, and Period

A descriptive cross-sectional study was conducted among patients visiting the public health centers of Gondar town. Gondar town is located in the Amhara regional state of Ethiopia, which is 727 km far from Addis Ababa. There are 8 health centers in Gondar town (Maraki, Mintewab, Gebrial, Azezo, Poly, Teda, Wolleka, and Bilajig). Gondar town has a total population of 454,445 based on the 2021 population estimation. The study was conducted from August to September 2021.

#### 2.1.1. Source Population

The source population was all patients who visit public health centers in Gondar town.

#### 2.1.2. Study Population

The study population was those patients who visit pharmacy units of public health centers of Gondar town during data collection.

### 2.2. Eligibility Criteria

Inclusion criteria were as follows: patients above 18 years old and attending the pharmacy dispensary unit and willing to participate in the study were included.

Exclusion criteria were as follows: patients with problems of cognition and communication were excluded from the study.

### 2.3. Sample Size Determination and Sampling Technique

The sample size was determined by using a single population proportion formula because the population is greater than 10,000 with the following assumption: 39.3% population (p) had unused/expired medicine at home according to a previous study [12], with a confidence interval 95%, and 5% of margin of error (d) estimates the total sample size.

(1) $$\begin{array}{c} =\frac{{\left(Z_{\sigma /2}\right)}^{2}p\left(1-p\right)}{d^{2}},\\ =\frac{{\left(1.96\right)}^{2}0.393\left(1-0.393\right)}{0.05^{2}}=367. \end{array}$$

**Figure 1:**
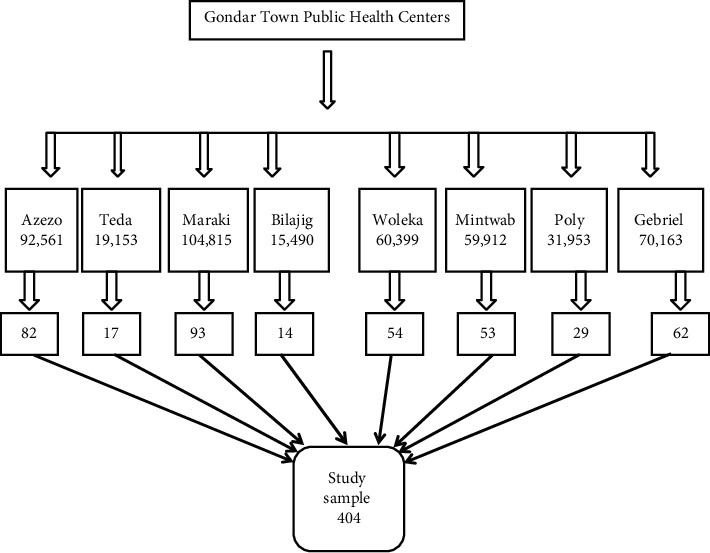
Schematic representation of the sampling procedure (*n* = 404).

For none responsive participants, 10% of the calculated sample size was taken and 404 become our final sample size. The health center was stratified based on their geographical location and patients were selected proportionally from each stratum by using a simple random sampling technique (Figure 1).

### 2.4. Data Collection Tool and Procedure

The data were collected by a face-to-face interview using a structured interview questionnaire. The questioner was prepared in consideration and reviewing other similar international literature [5, 7]. The tools used have four-section designed to address sociodemographic characteristics, knowledge, attitude, and practice of participants towards unused medications disposal. The final English version of the questionnaire was translated to Amharic language and translated back to English by independent professional translators to check the consistency. Furthermore, a pretest was conducted on the study population and important modifications were made accordingly. Finally, the data were collected by a graduating class pharmacy student after getting training regarding the data collection procedure, and the data collection process was supervised on daily basis.

### 2.5. Data Quality Control

To maintain data quality, an appropriate and valid data extraction tool was used. The data collection tool was pretested for consistency and completeness of data items on 5% of patients. For good consistency of data collection, the investigators and the adviser met three days before commencing the data collection to retrieve the data and to take a common understanding of the questioner.

### 2.6. Data Analysis

After the completion of data collection, the consistency and completeness of each questionnaire were assessed and coded before data entry into the software system. Then, the data were entered using the Epi-info version 7.2.2.6 and analyzed using the SPSS version 21.00. Descriptive statistics, such as frequency distribution, mean, and percentage, were employed for most variables.

### 2.7. Ethical Consideration

This research was conducted after ethical clearance was obtained from the school of pharmacy, University of Gondar, on Ref. No. SOP/272/2021. Verbal and written consent were taken from participants and health center managers' after explaining the objective and benefits of the study. The interview started after we got consent from the patients.

## 3. Result

### 3.1. Sociodemographic Characteristics of the Respondents

A total of 404 respondents participated in this study. The majority of them, 221 (54.7%), were female, and 232 (57.4%) were married. Most of them, 294 (72.8), were from urban areas, and 162 (40.1%) respondents had an educational status of college and above (Table 1).

### 3.2. Knowledge of Participants towards Disposal of Unused Medications

Among study participants, 286 (70.8%) of them knew about medication waste, and 149 (36.9%) of the respondents had not read or heard about instructions for unused medicine disposal practice. 357 (88.4%) participants knew that unused/expired medication causes harm when disposed of inappropriately, and 167 (41.7%) participants reported that disposing of unused medication inappropriately can be harmful to the environment. Accordingly, 266 (65.8%) participants assume that the hazardous effects of unused/expired medication could be reduced or minimized by providing proper guidance to the consumer. The respondents suggest that physicians, pharmacists, and the pharmaceutical industry are the responsible bodies for creating awareness in the community about the proper disposal of unused and expired medicines (Table 2).

### 3.3. Respondents' Attitude towards Disposal of Unused Medications

More than half (215 (53.5%)) of the participants strongly agreed that unused or expired medicine has a potential risk at home, and 170 (42.1%) of participants agreed that there is a lack of adequate information on the safe disposal of unused or expired medication. The majority of the respondents (279 (69.1)) strongly agreed that children are more vulnerable to the risk of unused or expired medicine at home. Above half (248 (61.4%)) of the respondents strongly agreed that doctors and healthcare professionals should provide the community with advice on the safe disposal of unused and expired medicine. One hundred seventy-one (42.3%) participants strongly agreed that an outreach and awareness program should be initiated to create awareness among people about how to dispose unused and expired medicine (Table 3).

### 3.4. Respondents Unused/Expired Medications Disposal Practice

More than half (55.9%) of the respondents had unused or expired medicine at home during the study period. The most common reasons for the respondents' having medication in their home were improvement of the medical conditions followed by a change in medication by a prescriber and intolerable side effects. Regarding disposal practice of unused medications, 150 (37.1%) of respondents throw their medications into household garbage, followed by a burn and flushed into the toilet sink. More than half (246 (60.9%)) of the participants discard their medication as it is, and 29 (7.2%) respondents reported that they do not know how to discard expired drugs (Table 4).

## 4. Discussion

Improper disposal practice of unused or expired medications is a danger for accidental poisoning and abuse, wasted resources, can kill aquatic or wildlife, and antibiotic resistance [6]. Based on the findings of the current study, 55.9% of the participants had unused medications at home. This finding is lower when compared to the study conducted in Indonesia, which revealed that 64.3% of the participants had unused medications at home [6]. Similarly, studies conducted in Saudi (79.1%) [9], Tanzania (70.19%) [13], and Dessie (70.19%) [4] reported that they had unused medications at home. However, the current finding is higher in a study conducted in the northern region of Ethiopia, in which 41.4% of respondents had unused medications at home [5], and also, a lower finding was reported in Gondar compressive specialized hospital which was 39.3% [14]. The observed difference might be due to a lack of awareness on what to do when medication is left over for different reasons in their homes. storing unused medication at home can have serious economical as well as medical consequences.

According to the findings of the current study, 127 (31.4%) of the respondents kept their medications at home because of improvement of the medical condition followed by intolerable side effects and intentions for future use. Their reasoning was similar to the study conducted in Dessie, Ethiopia [4], medical condition improvement 56.65% and change of medication by prescriber 23.57%. This may be due to a lack of information and proper advice by healthcare professionals on safe disposal practices for unused medications. Based on the findings of the current study, there were common disposal practices of unused medications. Most of the respondents threw their medications into household garbage followed by flushing the medication down the toilet/sink and keeping them at home. This finding was comparable to the study conducted in Indonesia [6], Saudi Arabia [9], UOG [14], and Tanzania [13]. This may be a lack of awareness about how to properly dispose unused medication and a difference in education programs.

Based on the findings of the current study, more than half 215 (53.5%) of the respondents strongly agreed that keeping unused medications at home can cause a potentially harmful risk. This finding was higher than a study conducted in Kenya which was 31.77% [15]. The observed difference might be due to a lack of educational programs and awareness. Based on the findings of the current study, 279 (69.1%) of respondents strongly agreed that children are more vulnerable to the potential risk of unused/expired medicine at home. This finding was relatively lower than the study conducted in Zambia, where 85.2% of the respondents strongly agreed [8], but the current finding was higher than the findings of Kenya, which was 32.32% [15]. The observed difference might be due to a lack of access to information or a difference in education about the disposal practices of unused medicine.

Among a total of 404 respondents, 170 (42.1%) of them agreed that there was no adequate information on the safe disposal of unused/expired medicine, and this was found to be lower when we were compared with a study conducted in Zambia, which found that there was 51.7% [8]. The observed difference might be due to a lack of communication between healthcare professionals and communities. In the current study, 171 (42.6%) and 161 (40.6%) of participants strongly agreed and agreed, respectively, about the need for the initiation of an outreach and awareness program to the community on how to dispose unused/expired medicines properly. This result was in line with a study done in the northern parts of Ethiopia [5]. This indicates that the participants have awareness of the initiation of the outreach program, and this suggestion could reduce medication wastage.

Based on the findings of the current study, 357 (88.4%) of the participants believed that there was a negative consequence because of the improper disposal of unused/expired medications into the environment. Accordingly, 171 (41.7%), 110 (27.2%), and 285 (70.5%) of participants believed that improper disposal of drugs can contaminate the environment, kill wildlife, and cause accidental swallowing by children, respectively, and they suggest that the harm could be minimized by providing proper guidance to the consumer 266 (65.8%) and prescribing in quantities and duration that ensure the patients' compliance 102 (25.2%). This result was relatively similar to a study conducted in Malaysia in which they reported that harm could be minimized by providing proper guidance 312 (73.2%) and prescribing in quantities that ensure the patients' compliance 114 (26.7%) [16].

The Food and Drug Administration recommends the preferred methods of disposal are take-back programs, returning to a nearby pharmacy, and appropriate household disposal methods. But the respondents from different studies did not employ these FDA methods, Indonesia [6], Tanzania [13], UOG, Ethiopia [14], Kuwait [17], and Dessie [4]. Similarly, in the current study, most respondents reported that they did not follow the FDA disposal method. This might be due to the unavailability of the facility for the take-back program or the lack of information about the recommended FDA disposal method.

About 29 (7.2%) respondents reported that they did not know what to do with expired medicine. This finding was comparable to the study done in Kuwait, which found 8% [17]. This might be due to a lack of education programs regarding the disposal of unused or expired medications. About, 272 (67.3%) respondents reported that they separate unused medications before disposal. This finding was relatively higher when compared to the study conducted in Mekele, 38 (62%) [5]. This might be due to different participants' awareness of unused medications.

### 4.1. Limitation of the Study

The study was conducted among patients visiting health centers; these might result in discrepancies with practices within the community. As the study is cross-sectional and depends on self-reported assessment, underreporting is more likely to occur.

## 5. Conclusion

Based on the findings of the current study, we concluded that there was a high practice of storage of unused/expired medication in households and almost all of the respondents did not follow the FDA-recommended safe disposal practice of unused medication. These can be sources of environmental hazards and public health problems.

## Figures and Tables

**Table 1:** Sociodemographic characteristics of respondents in public health centers of Gondar town, Northwest Ethiopia, September 2021 (*n* = 404).

Variables	Category	Frequency, *n* (%)
Gender	Male	183 (45.3)
	Female	221 (54.7)

Age (year)	18–24	105 (25.9)
	25–35	189 (46.7)
	36 and above	110 (27.2)

Marital status	Single	141 (34.9)
	Married	232 (57.4)
	Divorced	13 (3.2)
	Widowed	18 (4.5)

Residency	Rural	110 (27.2)
	Urban	294 (72.8)

Educational status	Unable to write and read	78 (19.3)
	Elementary school (1–8)	72 (17.8)
	Secondary (9–12)	92 (22.8)
	College and above	162 (40.1)

Occupations	Self-employed	156 (38.6)
	Governmental employee	88 (21.8)
	Student	100 (24.8)
	Housewife	60 (14.9)

**Table 2:** Knowledge of respondents about unused medicines disposal in public health centers of Gondar town, Northwest Ethiopia, September 2021 (*n* = 404).

Variables	Frequency (%)
Do you know about medication waste?	
Yes	286 (70.8)
No	118 (29.2)

If yes which one of the following can be considered as medicines waste?	
Expired medicines	188 (65.7)
Leftover medicines due to some reasons	114 (39.8)
Damaged medicines that cannot be used	108 (37.7)
Once opened medication and beyond their recommended day of use	89 (31.1)

Did you check the expiry date of your medication?	
Yes	239 (59.2)
No	165 (40.8)

Do you ever read or hear medicines disposal instructions?	
Yes	255 (63.1)
No	149 (36.7)

Do you know that inappropriate unused medicines disposal can cause harm?	
Yes	357 (88.4)
No	47 (11.6)

What could be the possible harm associated with inappropriate medicine disposal?	
It can contaminate the environment	167 (41.7)
It can kill wildlife	110 (27.2)
Can cause accidental swallow by children	285 (70.5)
Others	5 (1.23)

How could be the hazardous effect of unused and expired medicines minimized or controlled?	
Providing proper guidance to the consumer	266 (65.8)
Prescribing in quantities and for a duration that ensures patient compliance	102 (25.2)
Lowering the number of prescribed medicines by doctors	39 (9.7)
Donating or sharing the unused medicines	27 (6.7)
Others	2 (0.49)

Who is responsible to create awareness for the community about the proper disposal of unused and expired medicines?	
Mass media	95 (23.5)
Physician	245 (60.6)
Pharmacy	171 (42.3)
The pharmaceutical industry	91 (22.5)
Others	6 (1.48)

**Table 3:** Respondent's attitude towards disposal of unused medications at public health centers of Gondar town, September 2012 (*n* = 404).

Variable frequency, *n* (%)	
Do unused medicines present potential risks at home?	
Strongly agree	215 (53.5)
Agree	160 (39.6)
Neutral	16 (4)
Disagree	13 (3.2)

There is a lack of adequate information on the safe disposal of unused medicine	
Strongly agree	142 (35.1)
Agree	170 (42.1)
Neutral	33 (8.2)
Disagree	55 (13.5)
Strongly disagree	4 (1)

Children are more vulnerable to the risks associated with unused and expired household medicines	
Strongly agree	279 (69.1)
Agree	104 (25.7)
Neutral	10 (2.5)
Disagree	9 (2.2)
Strongly disagree	2 (0.5)

Doctors and healthcare professionals do provide advice on safe disposal of unused and expired household medicines	
Strongly agree	248 (61.8)
Agree	142 (35.1)
Neutral	8 (2)
Disagree	6 (1.5)

Take-back programs of unused and expired medicines should be mandatory	
Strongly agree	85 (21.3)
Agree	95 (23.5)
Neutral	74 (18.3)
Disagree	116 (28.7)
Strongly disagree	33 (8.2)

Outreach and awareness programs about how to dispose of unused or expired medicines should be initiated medicines	
Strongly agree	171 (42.3)
Agree	161 (40.6)
Neutral	24 (5.9)
Disagree	32 (7.9)
Strongly disagree	13 (3.2)

**Table 4:** Unused medicine disposal practice of respondents in public health centers of Gondar town, Northwest Ethiopia, September 2021 (*n* = 404).

Variable	Frequency, *n* (%)
Do you or anyone in your household have medications that are no longer needed?	
Yes	226 (55.9)
No	178 (44.1)

If yes, what is the reason for having unused medication?	
Improvement in medical condition	128 (56.7)
Change of medication by a prescriber	42 (18.6)
Intolerable side effect	34 (15.0)
Keeping for future use	22 (9.7)

What do you do with the unused medicines?	
Throw away in household garbage	150 (37.1)
Flush unused medications in toilet/sink	88 (21.8)
Keep at home until expired	28 (6.9)
Burn	101 (25)
Give to friends or relatives	15 (3.7)
Return to pharmacy	12 (3)
I do not know what to do	10 (2.5)

What do you do with the expired medicines?	
Throw away in household garbage	114 (28.2)
Flush unused medications in the toilet/sink	124 (30.7)
Keep at home until expired	16 (4)
Burn	111 (27.5)
Give to friends or relatives	2 (0.5)
Return to pharmacy	19 (4.7)
I do not know what to do	18 (4.5)

Do you separate unused medicines before disposal?	
Yes	272 (67.3)
No	132 (32.7)

## Data Availability

The datasets generated and/or analyzed during the current study are not available in public due to the requirement of confidentiality upon which consent was secured from the study participants but are available from the corresponding author upon request.
